# Supplementary material for: Bayesian refinement of protein structures and ensembles against SAXS data using molecular dynamics
Source: PLoS Comput Biol. 2017 Oct 18;13(10):e1005800. doi: 10.1371/journal.pcbi.1005800 (PMC5662244; doi:10.1371/journal.pcbi.1005800)
Supplement: S1 Archive — (BZ2) [file pcbi.1005800.s011.tar.bz2 › gromacs_4.62_bayesian_saxs_refi/share/html/online/g_rdf.html]

g\_rdf


| g\_rdf Main Table of Contents |

**VERSION 4.6  
Sat 19 Jan 2013**

---

### Description

The structure of liquids can be studied by either neutron or X-ray
scattering. The most common way to describe liquid structure is by a
radial distribution function. However, this is not easy to obtain from
a scattering experiment.

g\_rdf calculates radial distribution functions in different ways.
The normal method is around a (set of) particle(s), the other methods
are around the center of mass of a set of particles (-com)
or to the closest particle in a set (-surf).
With all methods, the RDF can also be calculated around axes parallel
to the z-axis with option -xy.
With option -surf normalization can not be used.

The option -rdf sets the type of RDF to be computed.
Default is for atoms or particles, but one can also select center
of mass or geometry of molecules or residues. In all cases, only
the atoms in the index groups are taken into account.
For molecules and/or the center of mass option, a run input file
is required.
Weighting other than COM or COG can currently only be achieved
by providing a run input file with different masses.
Options -com and -surf also work in conjunction
with -rdf.

If a run input file is supplied (-s) and -rdf is set
to atom, exclusions defined
in that file are taken into account when calculating the RDF.
The option -cut is meant as an alternative way to avoid
intramolecular peaks in the RDF plot.
It is however better to supply a run input file with a higher number of
exclusions. For e.g. benzene a topology, setting nrexcl to 5
would eliminate all intramolecular contributions to the RDF.
Note that all atoms in the selected groups are used, also the ones
that don't have Lennard-Jones interactions.

Option -cn produces the cumulative number RDF,
i.e. the average number of particles within a distance r.

To bridge the gap between theory and experiment structure factors can
be computed (option -sq). The algorithm uses FFT, the grid
spacing of which is determined by option -grid.

### Files

| option | filename | type | description |
| --- | --- | --- | --- |
| **-f** | traj.xtc | Input | Trajectory: xtc trr trj gro g96 pdb cpt |
| **-s** | topol.tpr | Input, Opt. | Structure+mass(db): tpr tpb tpa gro g96 pdb |
| **-n** | index.ndx | Input, Opt. | Index file |
| **-d** | sfactor.dat | Input, Opt. | Generic data file |
| **-o** | rdf.xvg | Output, Opt. | xvgr/xmgr file |
| **-sq** | sq.xvg | Output, Opt. | xvgr/xmgr file |
| **-cn** | rdf\_cn.xvg | Output, Opt. | xvgr/xmgr file |
| **-hq** | hq.xvg | Output, Opt. | xvgr/xmgr file |

### Other options

| option | type | default | description |
| --- | --- | --- | --- |
| **-[no]h** | bool | no | Print help info and quit || **-[no]version** | bool | no | Print version info and quit || **-nice** | int | 19 | Set the nicelevel || **-b** | time | 0 | First frame (ps) to read from trajectory || **-e** | time | 0 | Last frame (ps) to read from trajectory || **-dt** | time | 0 | Only use frame when t MOD dt = first time (ps) || **-[no]w** | bool | no | View output .xvg, .xpm, .eps and .pdb files || **-xvg** | enum | xmgrace | xvg plot formatting: xmgrace, xmgr or none || **-bin** | real | 0.002 | Binwidth (nm) || **-[no]com** | bool | no | RDF with respect to the center of mass of first group || **-surf** | enum | no | RDF with respect to the surface of the first group: no, mol or res || **-rdf** | enum | atom | RDF type: atom, mol\_com, mol\_cog, res\_com or res\_cog || **-[no]pbc** | bool | yes | Use periodic boundary conditions for computing distances. Without PBC the maximum range will be three times the largest box edge. || **-[no]norm** | bool | yes | Normalize for volume and density || **-[no]xy** | bool | no | Use only the x and y components of the distance || **-cut** | real | 0 | Shortest distance (nm) to be considered || **-ng** | int | 1 | Number of secondary groups to compute RDFs around a central group || **-fade** | real | 0 | From this distance onwards the RDF is tranformed by g'(r) = 1 + [g(r)-1] exp(-(r/fade-1)^2 to make it go to 1 smoothly. If fade is 0.0 nothing is done. || **-nlevel** | int | 20 | Number of different colors in the diffraction image || **-startq** | real | 0 | Starting q (1/nm) || **-endq** | real | 60 | Ending q (1/nm) || **-energy** | real | 12 | Energy of the incoming X-ray (keV) |

---

http://www.gromacs.org  
gromacs@gromacs.org
